# The impact of COVID‐19 pandemic lockdown on smoking habits and lifestyle: A cross‐sectional study

**DOI:** 10.1002/hsr2.1392

**Published:** 2023-06-30

**Authors:** Almu'atasim Khamees, Sajeda Awadi, Shireen Rawashdeh, Muna Talafha, Mai Alzoubi, Walaa Almdallal, Sharaf al‐Eitan, Ahmad Saeed, Raed M. Al‐Zoubi, Mazhar Salim Al‐Zoubi

**Affiliations:** ^1^ Faculty of Medicine Yarmouk University Irbid Jordan; ^2^ Department of Surgery King Hussein Cancer Center Amman Jordan; ^3^ Surgical Research Section, Department of Surgery Hamad Medical Corporation Doha Qatar; ^4^ Department of Biomedical Sciences, College of Health Sciences, QU‐Health Qatar University Doha Qatar; ^5^ Department of Chemistry Jordan University of Science and Technology Irbid Jordan

**Keywords:** COVID‐19, lockdown, SARS‐CoV‐19, smoking, smoking cessation

## Abstract

**Background and Aims:**

Throughout the COVID‐19 lockdown, the resultant psychological disturbances led to more tobacco consumption and deteriorated smoking behaviors among smokers. In this study, we aimed to investigate the impact of the COVID‐19 pandemic on the smoking behaviors of the Jordanian population.

**Methods:**

A cross‐sectional online survey was designed using the Google Forms service and distributed by social media platforms. Responses were collected starting from November 12, 2020, until November 24, 2020.

**Results:**

A total of 2511 respondents completed the survey, 77.3% were females. Males were significantly smoking more than females (*p* < 0.0001). Smoking was significantly more common among respondents who were older than 18 years old, married, held master's and PhD degrees, and working in non‐health‐related fields (*p* < 0.0001). Participants who smoke were more likely to adopt an unhealthy lifestyle during the pandemic. Females who started smoking last year were 2.6‐fold more than males (*p* < 0.0001). We also noticed that there is a significant relationship between those who started smoking and are <18 years, living in a family consisting of seven members or more, being unemployed, having a diploma or bachelor's degree in a health‐related major, having no chronic illnesses, increasing of daily meals or night meals, almost daily sugar intake, starting to follow social media account concerning physical activity, exercising once or twice a week, and sleeping more hours per day since the beginning of the pandemic (*p* < 0.01).

**Conclusion:**

The results of our study showed that the lockdown had a significant impact on people's lifestyles including smoking habits. Most of our sample's smoker participants experienced a change in their smoking level mostly, an increase. While those who had a decrease in their smoking level experienced a somehow healthier lifestyle regarding nutrition and other aspects.

## Highlights

- Respondents started smoking (22.4%) during the COVID‐19 year, mostly among females and singles.
- Respondents increased their tobacco consumption (45.4%) during the pandemic while 25.7% tried to smoke less or quit smoking.
- Smokers were more likely to adopt an unhealthy lifestyle during the lockdown compared to nonsmokers.
- Respondents who had a decrease in their smoking level experienced a somehow healthier lifestyle regarding nutrition.
- Free time, stress, and psychological factors were the main reasons for changing their smoking habits during the COVID‐19 pandemic.

## 1 INTRODUCTION

The World Health Organization (WHO) declared the novel coronavirus infection (COVID‐19) pandemic (SARS‐CoV‐2) a global health crisis in January 2020.^1^, ^2^ To decelerate virus transmission, many governments globally restricted the activities of their population with the implementation of strict “lockdown” measures.^3^ Between March and May 2020, Jordan applied an emergency nationwide lockdown to limit the spread of infection.^4^ The protective measures implemented also aimed to stop new cases that entered from surrounding countries by closing both land and air borders.^5^ Although these limitations helped to contain the outbreak, they resulted in adverse effects that negatively impacted the everyday life of the population^6^ due to social distancing and lack of contact. Consequently, this led to significant changes in lifestyle and daily habits.^7^, ^8^, ^9^ The subsequent psychological issues as a result of the pandemic led to more tobacco consumption.^10^ This tendency may be based on some smokers’ belief that smoking contributes to an improvement in psychological symptoms: a lessening of depressive symptoms and an improvement in anxiety.^11^ Moreover, an association has been established between physical isolation and lack of company during the pandemic and an increase in the average amount of cigarettes being smoked per day.^12^

Tobacco smoking, which is considered an addiction, has various harmful effects on multiple body systems and contributes to pulmonary toxicity.^13^ According to Global Youth Tobacco Survey data in 2014, the number of Jordanian students who have ever smoked any form of tobacco was overall 44%.^14^ It is believed that the protective measures that limited the movement of the population as well as the financial impact of the pandemic may contribute to a reduction in tobacco consumption.^15^ In light of the impact of smoking on respiratory health, smokers may have a greater concern compared to nonsmokers concerning contracting a respiratory infection such as COVID‐19.^16^, ^17^

Given all the above, the COVID‐19 pandemic is having a significant impact on people's smoking behavior during the lockdown. Whether these impacts are positive or negative, the main purpose of this study is to summarize and discuss how smoking habits changed during the lockdown among a sample of the Jordanian population.

## 2 METHODOLOGY

### 2.1 Procedure and data collection

A cross‐sectional study was conducted between November 12, 2020, and November 24, 2020. The online survey was distributed via social media platforms such as Facebook and WhatsApp, and via the Yarmouk University email portal to all the university students. The study targeted Jordanian people from all age groups and social/educational backgrounds. The total number of participants was 2511. At the beginning of the survey, we guaranteed that the data collected would only be used for this study and no other purpose. Cochran's formula has been used to calculate the sample size with precision level ± 0.5, confidence level = 95%, and estimated proportion = 0.5. The sample size in the study population exceeded the estimated sample size.^18^

### 2.2 Inclusion and exclusion criteria

All people who were ordinarily resident in Jordan since the start of the pandemic were eligible to complete the survey in the Arabic language. All participants that gave online consent for participation were included in this study.

### 2.3 Data collection

The questionnaire was designed to involve multiple sections with a variety of questions. The questionnaire began by collecting socio‐demographic data of the participant: age, gender, marital status (single or married), educational level (high school diploma or still in school, diploma or bachelor's degree in a health‐related major, diploma or bachelor's degree in a non‐health‐related major and master's degree or PhD), occupation (health‐related field, non‐health‐related field or unemployed), place of residence (city, village or refugee camps). Also, we asked the participants if they have a chronic disease and performed any physical activity or not.

The questionnaire included questions about certain lifestyle parameters before and after the pandemic lockdown including diet, number of meals, activity and exercise, app downloads, or started to follow any social media accounts concerning physical activity and exercise. The questionnaire further aimed to ascertain how many hours of sleep the participant managed as well as their smoking status. The study population was asked if they are smokers or not (including tobacco cigarettes, e‐cigarettes, and water pipes), and how long they were smoking and if they had experienced any noticeable changes in smoking levels, whether an increase/decrease or no change and the factors that led to these changes. Likewise, we asked respondents, who noticed a change in their smoking habits whether an increase or a decrease in the frequency of smoking, to choose the suitable reasons for that change. The reasons were divided into health, social, and psychological factors, free time, and the general lockdown of cafés and public smoking lounges. The questionnaire was designed for this study according to the adopted parameters. The questionnaire was validated and pretested by peers of medical and statistics at the Faculty of Medicine and the Department of Statistics.

### 2.4 Statistical analyses

The data collected were analyzed using the Statistical Package for the Social Sciences (SPSS) version 26 (IBM SPSS Corp, SPSS Statistics ver. 26). The categorical variables were summarized as frequencies and proportions and were compared using the chi‐square test. For all analyses, a *p* value less than 0.05 was considered statistically significant.

## 3 RESULTS

### 3.1 Socio‐demographic characteristics

The questionnaire was completed by 2511 respondents from all 12 governates in Jordan. Table 1 shows the demographic features of the study population including 77.3% females. Age groups of the respondents showed that 1661 (66.1%) are in the 18‐35 years age group. The majority of the participants live in a city, 1873 (74.6%). While 1559 (62.1%) of the respondents are single, and 1316 (52.4%) live in a family consisting of 4–6 members. The respondents' educational level varies as the following: 1825 (72.7%) hold a diploma or bachelor's degree and 820 of them have a degree in a health‐related major. On the other hand, 69.5% of the respondents are not working. 2231 (88.8%) do not suffer from any chronic illnesses. 1103 (43.9%) perform physical activity, and 478 (19.0%) are smokers.

**Table 1:** Demographic characteristics of the participants (*n* = 2511).

**Age**	
Under 18 years	151 (6.0%)
18–35 years	1661 (66.2%)
Over 35 years	699 (27.8%)
**Gender**	
Male	570 (22.7%)
Female	1941 (77.3%)
**Social status**	
Single	1559 (62.1%)
Married	952 (37.9%)
**Number of household members**	
3 or less	416 (16.6%)
4–6	1316 (52.4%)
7 or more	779 (31.0%)
**Educational level**	
High school diploma or still in school	380 (15.1%)
Diploma or bachelor's degree in a health‐related major	820 (32.7%)
Diploma or bachelor's degree in non‐health‐related major	1005 (40.0%)
Master's degree or PhD	306 (12.2%)
**Occupation**	
health‐related field	196 (7.8%)
non‐health‐related field	570 (22.7%)
I do not work	1745 (69.5%)
**Place of residence**	
City	1873 (74.6%)
Village	615 (24.5%)
Refugee camp	23 (0.9%)
**Do you suffer from any chronic illnesses?**	
Yes	280 (11.2%)
No	2231 (88.8%)
**Do you currently perform any form of physical exercise?**	
Yes	1103 (43.9%)
No	1408 (56.1%)
**Do you currently smoke (regular cigarettes, electronic cigarettes, hookah, etc.)?**	
Yes	478 (19.0%)
No	2033 (81.0%)

### 3.2 Smoking status

In our sample, 245 males out of 570 were smokers (43.0%) in comparison with 233 females who were smokers out of 1941 (12.0%), meaning there is almost a fourfold likeliness that males smoke compared to females by (*p* value < 0.0001). Smoking was more common among respondents who were older than 18 years old and those who hold a higher educational degree (master's and PhD degrees). Of participants who work in non‐health‐related fields, 28.2% were smokers compared with 18.3% of those who work in health‐related fields (*p* value < 0.0001). During the pandemic, participants who smoke were more likely to have decreased their number of daytime meals (19.2%) than nonsmokers (15.4%) (*p* value = 0.001). On the other hand, 50.2% of smokers declared that they had an increased number of nighttime meals in comparison with 37.6% of nonsmokers (*p* value < 0.0001). We also found that respondents who smoked were more likely to adopt an unhealthy lifestyle during the pandemic, demonstrated by the high frequency of fast‐food intake (three to four times a week or almost daily; *p* value < 0.0001) and low frequency of fresh fruits and vegetable intake (once a week; *p* value = 0.003). Likewise, smokers were significantly associated with sleeping fewer hours since the beginning of the pandemic (*p* value = 0.001), living in a family consisting of three members or less (*p* value ≤ 0.0001), and having no change in their level of exercise (*p* value = 0.040; Table 2).

**Table 2:** Relationship between the smoking status of participants (since the start of the pandemic) and the other variables (*n* = 2511).

	Do you currently smoke (regular cigarettes, electronic cigarettes, hookah, etc.)?			*p* Value
	Yes	No	Total	
**Age**				
Under 18 years	9 (0.4%)	142 (5.7%)	151 (6.0%)	**<0.001**
18–35 years	330 (13.1%)	1331 (53.0%)	1661 (66.1%)	
Over 35 years	139 (5.5%)	560 (22.3%)	699 (27.8%)	
**Gender**				
Male	245 (9.8%)	325 (12.9%)	570 (22.7%)	**<0.001**
Female	233 (9.3%)	1708 (68.0%)	1941 (77.3%)	
**Social status**				
Single	275 (11.0%)	1284 (51.1%)	1559 (62.1%)	**0.023**
Married	203 (8.1%)	749 (29.8%)	952 (37.9%)	
**Number of household members**				
3 or less	109 (4.3%)	307 (12.2%)	416 (16.6%)	**<0.001**
4–6	269 (10.7%)	1047 (41.7%)	1316 (52.4%)	
7 or more	100 (4.0%)	679 (27.0%)	779 (31%)	
**Educational level**				
High school diploma or still in school	64 (2.5%)	316 (12.6%)	380 (15.1%)	**<0.001**
Diploma or bachelor's degree in a health‐related major	123 (4.9%)	697 (27.8%)	820 (32.7%)	
Diploma or bachelor's degree in non‐health‐related major	212 (8.4%)	793 (31.6%)	1005 (40.0%)	
Master's degree or PhD	79 (3.1%)	227 (9.0%)	306 (12.2%)	
**Occupation**				
Health‐related field	36 (1.4%)	160 (6.4%)	196 (7.8%)	**<0.001**
Non‐health‐related field	161 (6.4%)	409 (16.3%)	570 (22.7%)	
I do not work	281 (11.2%)	1464 (58.3%)	1745 (69.5%)	
**Place of residence**				
City	372 (14.8%)	1501 (59.8%)	1873 (74.6%)	0.196
Village	102 (4.1%)	513 (20.4%)	615 (24.5%)	
Refugee camp	4 (0.2%)	19 (0.8%)	23 (0.9%)	
**Do you suffer from any chronic illnesses?**				
Yes	56 (2.2%)	224 (8.9%)	280 (11.2%)	0.663
No	422 (16.8%)	1809 (72.0%)	2231 (88.8%)	
**Do you currently perform any form of physical exercise?**				
Yes	206 (8.2%)	897 (35.7%)	1103 (43.9%)	0.684
No	272 (10.8%)	1136 (45.2%)	1408 (56.1%)	
**Fast food intake (after the start of the pandemic)**				
Once a month or less	217 (8.6%)	1324 (52.7%)	1541 (61.4%)	**<0.001**
Once a week	159 (6.3%)	515 (20.5%)	674 (26.9%)	
Three to four times a week	70 (2.8%)	132 (5.3%)	202 (8.0%)	
Almost daily	32 (1.3%)	61 (2.4%)	93 (3.7%)	
**Sugar intake (after the start of the pandemic)**				
Once a month or less	46 (1.8%)	206 (8.2%)	252 (10.0%)	0.218
Once a week	111 (4.4%)	561 (22.3%)	672 (26.8%)	
Three to four times a week	157 (6.3%)	632 (25.2%)	789 (31.4%)	
Almost daily	164 (6.5%)	634 (25.2%)	798 (31.8%)	
**Fat and oil intake (after the start of the pandemic)**				
Once a month or less	41 (1.6%)	201 (8.0%)	242 (9.6%)	0.855
Once a week	109 (4.3%)	452 (18.0%)	561 (22.3%)	
Three to four times a week	175 (7.0%)	736 (29.3%)	911 (36.3%)	
Almost daily	153 (6.1%)	644 (25.6%)	797 (31.7%)	
**Fresh fruit and vegetable intake (after the start of the pandemic)**				
Once a month or less	20 (0.8%)	72 (2.9%)	92 (3.7%)	**0.003**
Once a week	69 (2.7%)	185 (7.4%)	254 (10.1%)	
Three to four times a week	138 (5.5%)	581 (23.1%)	719 (28.6%)	
Almost daily	251 (10.0%)	1194 (47.6%)	1445 (57.6%)	
**(Since the start of the pandemic) have you noticed any change in your weight?**				
I have gained weight	184 (7.3%)	729 (29.0%)	913 (36.4%)	0.282
I have lost weight	98 (3.9%)	375 (14.9%)	473 (18.8%)	
My weight has not changed	170 (6.8%)	791 (31.5%)	961 (38.3%)	
I do not know	26 (1.0%)	138 (5.5%)	164 (6.5%)	
**(Since the start of the pandemic) has the number of meals you eat in a day changed?**				
Increased	184 (7.3%)	660 (26.3%)	844 (33.6%)	**0.001**
Decreased	92 (3.7%)	315 (12.5%)	407 (16.2%)	
No change	202 (8.0%)	1058 (42.1%)	1260 (50.2%)	
**(Since the start of the pandemic) has the number of meals you eat during nighttime increased?**				
Yes	240 (9.6%)	765 (30.5%)	1005 (40.0%)	**<0.001**
No	238 (9.5%)	1268 (50.5%)	1506 (60.0%)	
**(Since the start of the pandemic) have you noticed any change in your appetite?**				
My appetite has increased	187 (7.4%)	758 (30.2%)	945 (37.6%)	0.236
My appetite has decreased	87 (3.5%)	325 (12.9%)	412 (16.4%)	
My appetite has not changed	204 (8.1%)	950 (37.8%)	1154 (46.0%)	
**Downloading any application or started following any social media account concerning Physical Activity and Exercise**				
Yes	168 (6.7%)	758 (30.2%)	926 (36.9%)	0.383
No	310 (12.3%)	1275 (50.8%)	1585 (63.1%)	
**During the pandemic, did you notice a change related to your utilization of different electronic devices (mobile phones, laptops, television, etc.)?**				
More	418 (16.6%)	1720 (68.5%)	2138 (85.1%)	0.193
Less	8 (0.3%)	29 (1.2%)	37 (1.5%)	
I did not notice any change	52 (2.1%)	284 (11.3%)	336 (13.4%)	
**Since the beginning of the pandemic, have you noticed a change with regard to your sleep hours per day?**				
I started sleeping less	77 (3.1%)	262 (10.4%)	339 (13.5%)	**0.001**
I started sleeping more	216 (8.6%)	786 (31.3%)	1002 (39.9%)	
No change	185 (7.4%)	985 (39.2%)	1170 (46.6%)	
**Total**	**478** (**19.0%)**	**2033** (**81.0%)**	**2511** (**100.0%)**	

*Note*: Values are expressed as number and percentage from total respondents (*n* (%)). Variables are considered significant at *p* value < 0.05 and marked in bold.

There was no significant difference with the place of residency, the presence of chronic disease, changes in weight, appetite or frequency of sugar, fat, and oil intake after the start of the pandemic, using electronic devices, exercise status, and frequency of exercise (*p* value for all > 0.05; Table 2).

### 3.3 Starting to smoke

When the respondents were asked about the starting of smoking, 107 out of 478 participants who smoke (22.4%) started smoking during the past year, 249 smoker participants (52.1%) started smoking 2–10 years ago, while 122 (25.5%) started smoking more than 10 years ago.

Among participants who started smoking last year, 31 out of 245 (12.7%) were males and 76 out of 233 (32.6%) were females, which means that females are more by 2.6‐fold. On the other hand, 87 out of 245 (35.5%) males while 35 out of 233 females (15.0%) started smoking before 10 years, making males more by 2.4‐fold (*p* value = < 0.0001). Moreover, 81 out of 275 (29.5%) single participants started smoking in the past year, while 26 out of 203 married participants (12.8%) started smoking in the last year, this means that single participants are more by 2.3‐fold. However, 20 out of 275 single participants (7.3%) started smoking more than 10 years while 102 out of 203 married participants (50.2%) started smoking more than 10 years so married participants are more by 6.9‐fold (*p* value ≤ 0.0001; Table 3).

**Table 3:** Relationship between the duration from starting smoking and the other variables (*n* = 478).

	When did you start smoking?				*p* Value
	During the past year	2–10 years ago	More than 10 years ago	Total	
**Age**					
Under 18 years	6 (1.3%)	3 (0.6%)	0 (0.0%)	9 (1.9%)	**<0.001**
18–35 years	88 (18.4%)	204 (42.7%)	38 (7.9%)	330 (69.0%)	
Over 35 years	13 (2.7%)	42 (8.8%)	84 (17.6%)	139 (29.1%)	
**Gender**					
Male	31 (6.5%)	127 (26.6%)	87 (18.2%)	245 (51.3%)	**<0.001**
Female	76 (159%)	122 (25.5%)	35 (7.3%)	233 (48.7%)	
**Social status**					
Single	81 (16.9%)	174 (36.4%)	20 (4.2%)	275 (57.5%)	**<0.001**
Married	26 (5.4%)	75 (15.7%)	102 (21.3%)	203 (42.5%)	
**Number of household members**					
3 or less	23 (4.8%)	58 (12.1%)	28 (5.9%)	109 (22.8%)	**0.001**
4–6	47 (9.8%)	144 (30.1%)	78 (16.3%)	269 (56.3%)	
7 or more	37 (7.7%)	47 (9.8%)	16 (3.3%)	100 (20.9%)	
**Educational level**					
High school diploma or still in school	16 (3.3%)	26 (5.4%)	22 (4.6%)	64 (13.4%)	**<0.001**
Diploma or bachelor's degree in a health‐related major	44 (9.2%)	69 (14.4%)	10 (2.1%)	123 (25.7%)	
Diploma or bachelor's degree in non‐health‐related major	43 (9.0%)	122 (25.5%)	47 (9.8%)	212 (44.4%)	
Master's degree or PhD	4 (0.8%)	32 (6.7%)	43 (9.0%)	79 (16.5%)	
**Occupation**					
Health‐related field	3 (0.6%)	20 (4.2%)	13 (2.7%)	36 (7.5%)	**<0.001**
Non‐health‐related field	23 (4.8%)	68 (14.2%)	70 (14.6%)	161 (33.7%)	
I do not work	81 (16.9%)	161 (33.7%)	39 (8.2%)	281 (58.8%)	
**Place of residence**					
City	80 (16.7%)	204 (42.7%)	88 (18.4%)	372 (77.8%)	0.150
Village	25 (5.2%)	44 (9.2%)	33 (6.9%)	102 (21.3%)	
Refugee camp	2 (0.4%)	1 (0.2%)	1 (0.2%)	4 (0.8%)	
**Do you suffer from any chronic illnesses?**					
Yes	9 (1.9%)	18 (3.8%)	29 (6.1%)	56 (11.7%)	**<0.001**
No	98 (20.5%)	231 (48.3%)	93 (19.5%)	422 (88.3%)	
**Do you currently perform any form of physical exercise?**					
Yes	45 (9.4%)	116 (24.3%)	45 (9.4%)	206 (43.1%)	0.202
No	62 (13.0%)	133 (27.8%)	77 (16.1%)	272 (56.9%)	
**Frequency of exercise**					
Irregularly	25 (12.1%)	51 (24.8%)	14 (6.8%)	90 (43.7%)	**0.006**
Once or twice a week	10 (4.9%)	15 (7.3%)	5 (2.4%)	30 (14.6%)	
Three to five times a week	4 (1.9%)	28 (13.6%)	8(3.9%)	40 (19.4%)	
Almost everyday	6 (2.9%)	22 (10.7%)	18 (8.7%)	46 (22.3%)	
**Have you noticed any change in your level of exercise or even a change in the way you think of exercise since the beginning of the pandemic?**					
No change	12 (5.8%)	21 (10.2%)	15 (7.3%)	48 (23.3%)	**0.023**
I have realized the importance of exercise but do not necessarily apply it to my daily routine	19 (9.2%)	38 (18.4%)	8 (3.9%)	65 (31.6%)	
I have started doing some form of exercise	10 (4.9%)	21 (10.2%)	10 (4.9%)	41 (19.9%)	
No change	4 (1.9%)	36 (17.5%)	12 (5.8%)	52 (25.2%)	
**Fast food intake (after the start of the pandemic)**					
Once a month or less	46 (9.6%)	106 (22.2%)	65 (13.6%)	217 (45.4%)	0.275
Once a week	38 (7.9%)	82 (17.2%)	39 (8.2%)	159 (33.3%)	
Three to four times a week	17 (3.6%)	43 (9.0%)	10 (2.1%)	70 (14.6%)	
Almost daily	6 (1.3%)	18 (3.8%)	8 (1.7%)	32 (6.7%)	
**Sugar intake (after the start of the pandemic)**					
Once a month or less	6 (1.3%)	20 (4.2%)	20 (4.2%)	46 (9.6%)	**0.006**
Once a week	18 (3.8%)	56 (11.7%)	37 (7.7%)	111 (23.2%)	
Three to four times a week	40 (8.4%)	85 (17.8%)	32 (6.7%)	157 (32.8%)	
Almost daily	43 (9.0%)	88 (18.4%)	33 (6.9%)	164 (34.3%)	
**Fat and oil intake (after the start of the pandemic)**					
Once a month or less	6 (1.3%)	21 (4.4%)	14 (2.9%)	41 (8.6%)	0.248
Once a week	23 (4.8%)	51 (10.7%)	35 (7.3%)	109 (22.8%)	
Three to four times a week	38 (7.9%)	95 (19.9%)	42 (8.8%)	175 (36.6%)	
Almost daily	40 (8.4%)	82 (17.2%)	31 (6.5%)	153 (32.0%)	
**Fresh fruit and vegetable intake (after the start of the pandemic)**					
Once a month or less	8 (1.7%)	7 (1.5%)	5 (1.0%)	20 (4.2%)	0.321
Once a week	18 (3.8%)	38 (7.9%)	13 (2.7%)	69 (14.4%)	
Three to four times a week	26 (5.4%)	76 (15.9%)	36 (7.5%)	138 (28.9%)	
Almost daily	55 (11.5%)	128 (26.8%)	68 (14.2%)	251 (52.5%)	
**(Since the start of the pandemic) have you noticed any change in your weight?**					
I have gained weight	48 (10.0%)	95 (19.9%)	41 (8.6%)	184 (38.5%)	0.122
I have lost weight	24 (5.0%)	55 (11.5%)	19 (4.0%)	98 (20.5%)	
My weight has not changed	28 (5.9%)	87 (18.2%)	55 (11.5%)	170 (35.6%)	
I do not know	7 (1.5%)	12 (2.5%)	7 (1.5%)	26 (5.4%)	
**(Since the start of the pandemic) has the number of meals you eat in a day changed?**					
Increased	54 (11.3%)	95 (19.9%)	35 (7.3%)	184 (38.5%)	**0.001**
Decreased	23 (4.8%)	49 (10.3%)	20 (4.2%)	92 (19.2%)	
No change	30 (6.3%)	105 (22.0%)	67 (14.0%)	202 (42.3%)	
**(Since the start of the pandemic) has the number of meals you eat during nighttime increased?**					
Yes	68 (14.2%)	120 (25.1%)	52 (10.9%)	240 (50.2%)	**0.004**
No	39 (8.2%)	129 (27.0%)	70 (14.6%)	238 (49.8%)	
**(Since the start of the pandemic) have you noticed any change in your appetite?**					
Increased	53 (11.1%)	94 (19.7%)	40 (8.4%)	187 (39.1%)	0.063
Decreased	18 (3.8%)	49 (10.3%)	20 (4.2%)	87 (18.2%)	
No change	36 (7.5%)	106 (22.2%)	62 (13.0%)	204 (42.7%)	
**Downloading any application or started following any social media account concerning Physical Activity and Exercise**					
Yes	48 (10.0%)	92 (19.2%)	28 (5.9%)	168 (35.1%)	**0.002**
No	59 (12.3%)	157 (32.8%)	94 (19.7%)	310 (64.9%)	
**During the pandemic, did you notice a change related to your utilization of different electronic devices (mobile phones, laptops, television, etc.)?**					
Using more	96 (20.1%)	222 (46.4%)	100 (20.9%)	418 (87.4%)	0.151
Using less	3 (0.6%)	2 (0.4%)	3 (0.6%)	8 (1.7%)	
No change	8 (1.7%)	25 (5.2%)	19 (4.0%)	52 (10.9%)	
**Since the beginning of the pandemic, have you noticed a change with regard to your sleep hours per day?**					
Less	17 (3.6%)	47 (9.8%)	13 (2.7%)	77 (16.1%)	**0.010**
More	60 (12.6%)	106 (22.2%)	50 (10.5%)	216 (45.2%)	
No change	30 (6.3%)	96 (20.1%)	59 (12.3%)	185 (38.7%)	
**Since the beginning of the pandemic, have you noticed any change related to your smoking habits?**					
I started smoking more	42 (8.8%)	109 (22.8%)	66 (13.8%)	217 (45.4%)	0.092
I started/tried smoking less or tried to quit	32 (6.7%)	70 (14.6%)	21 (4.4%)	123 (25.7%)	
I have not noticed a change	33 (6.9%)	70 (14.6%)	35 (7.3%)	138 (28.9%)	
	**107** (**22.4%)**	**249** (**52.1%)**	**122** (**25.5%)**	**478** (**100.0%)**	

*Note*: Values are expressed as number and percentage from total respondents (*n* (%)). Variables are considered significant at *p* value < 0.05 and marked in bold.

We also noticed that respondents who started smoking during the last year were more likely to be those who had a diploma or bachelor's degree in a health‐related major (35.8%) This is compared to those with a high school diploma or still in school (25.0%), diploma or bachelor's degree in non‐health‐related major (20.3%), and master's degree or PhD (5.1%; *p* value ≤ 0.0001). While respondents who started smoking more than 10 years ago were more likely to be found those who had a master's degree or PhD (54.4%) than a high school diploma or still in school (34.4%), diploma, or bachelor's degree in non‐health‐related major (22.2%), and diploma or bachelor's degree in a health‐related major (8.1%; *p* value ≤ 0.0001).

We also noticed that there is a significant relationship between those starting to smoke in the last year being under 18 years old (*p* value ≤ 0.0001), living in a family consisting of seven members or more (*p* value = 0.001), unemployed (*p* value = 0.000), having a diploma or bachelor's degree in a health‐related major (*p* value ≤ 0.0001), having no chronic illnesses (*p* value ≤ 0.0001), increasing the number of daily meals (*p* value = 0.001) or night meals (*p* value = 0.004), starting to follow social media account concerning physical activity (*p* value = 0.002), doing the exercise once or twice a week (*p* value = 0.006), and sleeping more hours per day since the beginning of the pandemic (*p* value = 0.01; Table 3).

Regarding sugar consumption after the pandemic, among those who started smoking in the past year, 26.2% declared that they had a daily sugar consumption, 25.5% had three to four times a week sugar consumption, 16.2% had once‐a‐week sugar consumption and 13% had once monthly or less sugar consumption. However, 35.3% of those who started smoking 2–10 years ago had almost daily sugar consumption after the pandemic, 34.1% had three to four times a week sugar consumption, 22.4% had once‐a‐week sugar consumption, 8% had once‐a‐month or less sugar consumption (*p* value = 0.006).

However, there was no significant difference between duration since starting smoking and place of residency, physical exercise performance, change in appetite, change in weight, fast food intake, fat and oil intake, fresh fruit and vegetable intake (*p* value for all >0.05; Table 3).

### 3.4 Changing related to the smoking habits

**Figure 1:**
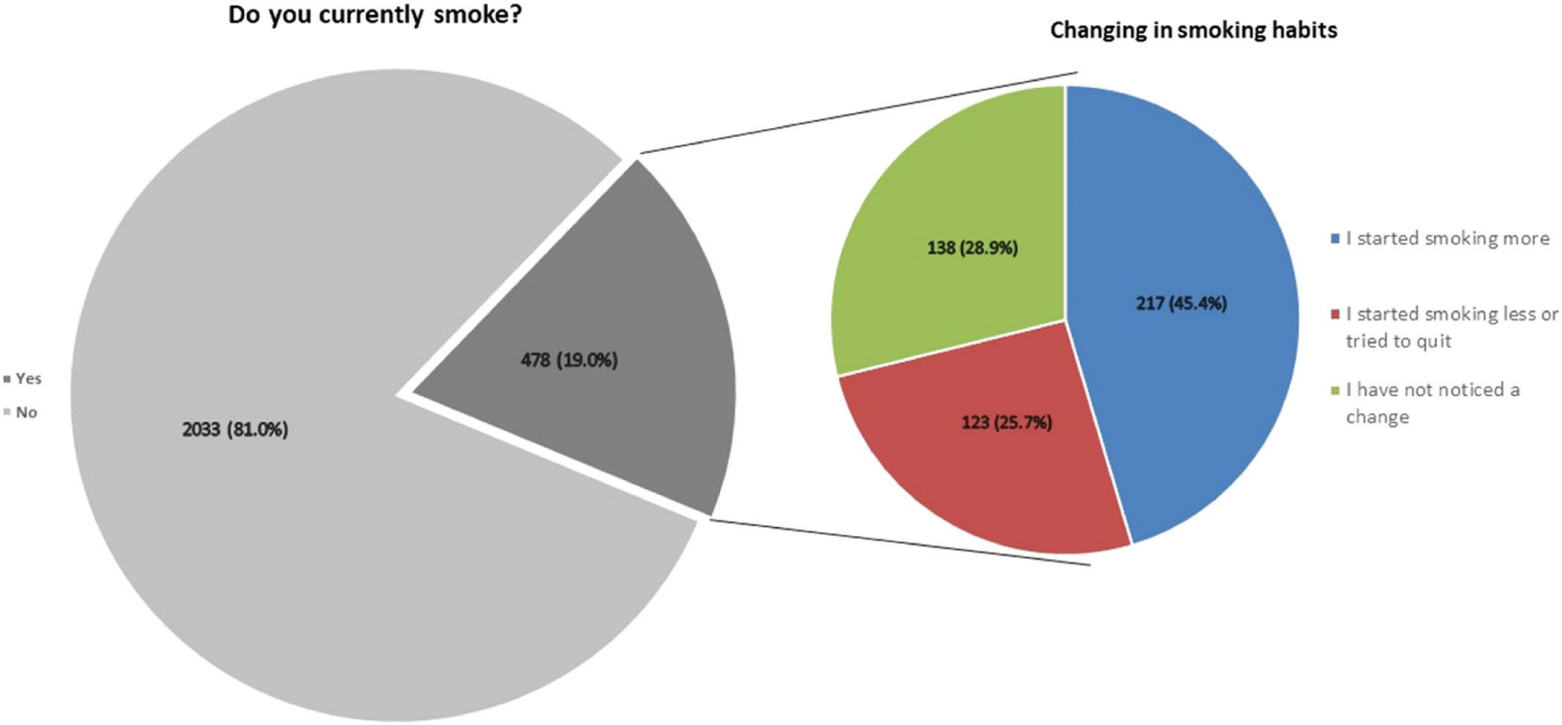
Smoking status (regular cigarettes, electronic cigarettes, hookah, etc.), and changes in smoking habits among smokers since the beginning of the pandemic.

Two hundred seventeen out of 478 smoker participants (45.4%) stated they had increased their tobacco consumption during the pandemic, 123 smoker participants (25.7%) tried to smoke less or quit smoking during the pandemic while 138 smoker participants (28.9%) noticed no change in their smoking level during the pandemic (Figure 1).

Regarding smoking habits during the pandemic, 75.6% of participants who declared that they smoked less were mostly in the (18–35 years) age group. We noticed that most people who stated they were smoking less were more likely to download apps or follow social media accounts concerning physical activity and Exercise (47.2%). They also tended to have a decrease in their appetite (20.3%), to lose weight (24.4%), and to have no increase in the number of night meals (51.2%; *p* value for all were less than 0.05; Table 4).

**Table 4:** Relationship between changes in smoking habits since the beginning of pandemic and the other variables (*n* = 478).

	Since the beginning of the pandemic, have you noticed any change related to your smoking habits?				*p* Value
	I started smoking more	I started/tried smoking less or tried to quit	I have not noticed a change	Total	
**Age**					
Under 18 years	4 (0.8%)	1 (0.2%)	4 (0.8%)	9 (1.9%)	**0.010**
18–35 years	158 (33.1%)	93 (19.5%)	79 (16.5%)	330 (69.0%)	
Over 35 years	55 (11.5%)	29 (6.1%)	55 (11.5%)	139 (29.1%)	
**Gender**					
Male	121 (25.3%)	58 (12.1%)	66 (13.8%)	245 (51.3%)	0.198
Female	96 (20.1%)	65 (913.6%)	72 (15.1%)	233 (48.7%)	
**Social status**					
Single	130 (27.2%)	71 (14.9%)	74 (15.5%)	275 (57.5%)	0.505
Married	87 (18.2%)	52 (10.9%)	64 (13.4%)	203 (42.5%)	
**Number of household members**					
3 or less	48 (10.0%)	34 (7.1%)	27 (5.6%)	109 (22.8%)	0.072
4–6	131 (27.4%)	56 (11.7%)	82 (17.2%)	269 (56.3%)	
7 or more	38 (97.9%)	33 (6.9%)	29 (6.1%)	100 (20.9%)	
**Educational level**					
High school diploma or still in school	29 (6.1%)	15 (3.1%)	20 (4.2%)	64 (13.4%)	0.152
Diploma or bachelor's degree in a health‐related major	48 (10.0%)	43 (9.0%)	32 (6.7%)	123 (25.7%)	
Diploma or bachelor's degree in non‐health‐related major	97 (20.3%)	49(10.3%)	66 (13.8%)	212 (44.4%)	
Master's degree or PhD	43 (9.0%)	16 (3.3%)	20 (4.2%)	79 (16.5%)	
**Occupation**					
Health‐related field	13 (2.7%)	12 (2.5%)	11 (2.3%)	36 (7.5%)	0.600
Non‐health‐related field	72 (15.1%)	38 (7.9%)	51 (10.7%)	161 (33.7%)	
I do not work	132 (27.6%)	73 (15.3%)	76 (15.9%)	281 (58.8%)	
**Place of residence**					
City	170 (35.6%)	98 (20.5%)	104 (21.8%)	372 (77.8%)	0.708
Village	45 (9.4%)	25 (5.2%)	32 (6.7%)	102 (21.3%)	
Refugee camp	2 (0.4%)	0 (0.0%)	2 (0.4%)	4 (0.8%)	
**Do you suffer from any chronic illnesses?**					
Yes	27 (5.6%)	14 (2.9%)	15 (3.1%)	56 (11.7%)	0.896
No	190 (39.7%)	109 (22.8%)	123 (25.7%)	422 (88.3%)	
**Do you currently perform any form of physical exercise?**					
Yes	89 (18.6%)	64 (13.4%)	53 (11.1%)	206 (43.1%)	0.060
No	128 (26.8%)	59 (12.3%)	85 (17.8%)	272 (56.9%)	
**Frequency of exercise**					
Irregularly	38 (18.4%)	30 (14.6%)	22 (10.7%)	90 (43.7%)	0.524
Once or twice a week	14 (6.8%)	11 (5.3%)	5 (2.4%)	30 (14.6%)	
Three to five times a week	15 (7.3%)	14 (6.8%)	11 (5.3%)	40 (19.4%)	
Almost everyday	22 (10.7%)	9 (4.4%)	15 (7.3%)	46 (22.3%)	
**Have you noticed any change in your level of exercise or even a change in the way you think of exercise since the beginning of the pandemic?**					
No change	21 (10.2%)	10 (4.9%)	17 (8.3%)	48 (23.3%)	0.287
I have realized the importance of exercise but do not necessarily apply it to my daily routine	28 (13.6%)	25 (12.1%)	12 (5.8%)	65 (31.6%)	
I have started doing some form of exercise.	19 (9.2%)	14 (6.8%)	8 (3.9%)	41 (19.9%)	
No change	21 (10.2%)	15 (7.3%)	16 (7.8%)	52 (25.2%)	
**Fast food intake (after the start of the pandemic)**					
Once a month or less	95 (19.9%)	52 (10.9%)	70 (14.6%)	217 (45.4%)	**0.044**
Once a week	63 (13.2%)	52 (10.9%)	44 (9.2%)	159 (33.3%)	
Three to four times a week	39 (8.2%)	15 (3.1%)	16 (3.3%)	70 (14.6%)	
Almost daily	20 (94.2%)	4 (0.8%)	8 (1.7%)	32 (6.7%)	
**Sugar intake (after the start of the pandemic)**					
Once a month or less	17 (3.6%)	13 (2.7%)	16 (3.3%)	46 (9.6%)	**0.038**
Once a week	51 (10.7%)	32 (6.7%)	28 (5.9%)	111 (23.2%)	
Three to four times a week	59 (12.3%)	48 (10.0%)	50 (10.5%)	157 (32.8%)	
Almost daily	90 (18.8%)	30 (6.3%)	44 (9.2%)	164 (34.3%)	
**Fat and oil intake (after the start of the pandemic)**					
Once a month or less	18 (3.8%)	12 (2.5%)	11 (2.3%)	41 (8.6%)	0.207
Once a week	46 (9.6%)	31 (6.5%)	32 (6.7%)	109 (22.8%)	
Three to four times a week	71 (14.9%)	52 (10.9%)	52 (10.9%)	175 (36.6%)	
Almost daily	82 (17.2%)	28 (5.9%)	43 (9.0%)	153 (32.0%)	
**Fresh fruit and vegetable intake (after the start of the pandemic)**					
Once a month or less	9 (1.9%)	4 (0.8%)	7 (1.5%)	20 (4.2%)	0.698
Once a week	34 (7.1%)	16 (3.3%)	19 (4.0%)	69 (14.4%)	
Three to four times a week	54 (11.3%)	41 (8.6%)	43 (9.0%)	138 (28.9%)	
Almost daily	120 (25.1%)	62 (13.0%)	69 (14.4%)	251 (52.5%)	
**(Since the start of the pandemic) have you noticed any change in your weight?**					
I have gained weight	89 (18.6%)	54 (11.3%)	41 (8.6%)	184 (38.5%)	**0.009**
I have lost weight	45 (9.4%)	30 (6.3%)	23 (4.8%)	98 (20.5%)	
My weight has not changed	68 (14.2%)	36 (7.5%)	66 (13.8%)	170(35.6%)	
I do not know	15 (3.1%)	3 (0.6%)	8 (1.7%)	26 (5.4%)	
**(Since the start of the pandemic) has the number of meals you eat in a day changed?**					
Increased	94 (19.7%)	50 (10.5%)	40 (8.4%)	184 (38.5%)	0.053
Decreased	42 (8.8%)	24 (5.0%)	26 (5.4%)	92 (19.2%)	
No change	81 (16.9%)	49 (10.3%)	72 (15.1%)	202 (42.3%)	
**(Since the start of the pandemic) has the number of meals you eat during nighttime increased?**					
Yes	129 (27.0%)	60 (12.6%)	51 (10.7%)	240 (50.2%)	**<0.001**
No	88 (18.4%)	63 (13.2%)	87 (18.2%)	238 (49.8%)	
**(Since the start of the pandemic) have you noticed any change in your appetite?**					
Increased	105 (22.0%)	43 (9.0%)	39 (8.2%)	187 (39.1%)	**0.002**
Decreased	36 (7.5%)	25 (5.2%)	26 (5.4%)	87 (18.2%)	
No change	76 (15.9%)	55 (11.5%)	73 (15.3%)	204 (42.7%)	
**Downloading any application or started following any social media account concerning Physical Activity and Exercise**					
Yes	74 (15.5%)	58 (12.1%)	36 (7.5%)	168 (35.1%)	**0.002**
No	143 (29.9%)	65 (13.6%)	102 (21.3%)	310 (64.9%)	
**During the pandemic, did you notice a change related to your utilization of different electronic devices (mobile phones, laptops, television, etc.)?**					
Using more	202 (42.3%)	107 (22.4%)	109 (22.8%)	418 (87.4%)	**0.003**
Using less	1 (0.2%)	3 (0.6%)	4 (0.8%)	8 (1.7%)	
No change	14 (2.9%)	13 (2.7%)	25 (5.2%)	52 (10.9%)	
**During the pandemic, how many hours do you sleep per day?**					
Less than 7 h	52 (10.9%)	25 (5.2%)	33 (6.9%)	110 (23.0%)	**0.009**
7–9 h	104 (21.8%)	73 (15.3%)	87 (18.2%)	264 (55.2%)	
More than 9 h	61 (12.8%)	25 (5.2%)	18 (3.8%)	104 (21.8%)	
**Since the beginning of the pandemic, have you noticed a change with regard to your sleep hours per day?**					
Less	35 (7.3%)	21 (4.4%)	21 (4.4%)	77 (16.1%)	**<0.001**
More	128 (26.8%)	54 (11.3%)	34 (7.1%)	216 (45.2%)	
No change	54 (11.3%)	48 (10.0%)	83 (17.4%)	185 (38.7%)	
**When did you start smoking?**					
During the past year	42 (8.8%)	32 (6.7%)	33 (6.9%)	107 (22.4%)	0.092
2–10 years ago	109 (22.8%)	70 (14.6%)	70 (14.6%)	249 (52.1%)	
More than 10 years ago	66 (13.8%)	21 (4.4%)	35 (7.3%)	122 (25.5%)	
**Total**	**217** (**45.4%)**	**123** (**25.7%)**	**138** (**28.9%)**	**478** (**100.0%)**	

*Note*: Values are expressed as number and percentage from total respondents (*n* (%)). Variables are considered significant at *p*‐value < 0.05 and marked in bold.

On the other hand, those who stated they were smokers and were smoking more during the pandemic were: also mostly in the (18–35) age group (72.8%), less likely to download apps or follow social media accounts concerning physical activity and exercise (65.8%), to have an increase in their appetite (48.3%), to gain weight (41.0%), and to have an increase in the number of night meals (59.4%) and to sleep more hours during the pandemic (58.9%; *p* value for all were less than 0.05).

Those who smoked more during the pandemic declared that their fast‐food intake was as follows: almost daily (62.5%), three to four times a week (55.7%), once monthly or less (43.8%), once a week (39.6%). While those who declared smoking less or tried to quit during the pandemic their fast‐food intake was as follows: once a week (32.7%), once monthly or less (24.0%), three to four times a week (21.4%), almost daily (12.5%) (*p* value = 0.04).

Regarding sugar intake during the pandemic, those who smoked more during the pandemic declared that their sugar intake was as follows: almost daily (54.9%), once a week (45.9%), three to four times a week (37.6%), once monthly or less sugar consumption (37.0%). However, for those who declared smoking less or tried to quit during the pandemic their sugar intake was as follows: three to four times a week (30.6%), once a week (28.8%), once monthly or less sugar consumption (28.3%), almost daily (18.3%; *p* value = 0.038).

### 3.5 Reasons for changing in smoking habits

**Figure 2:**
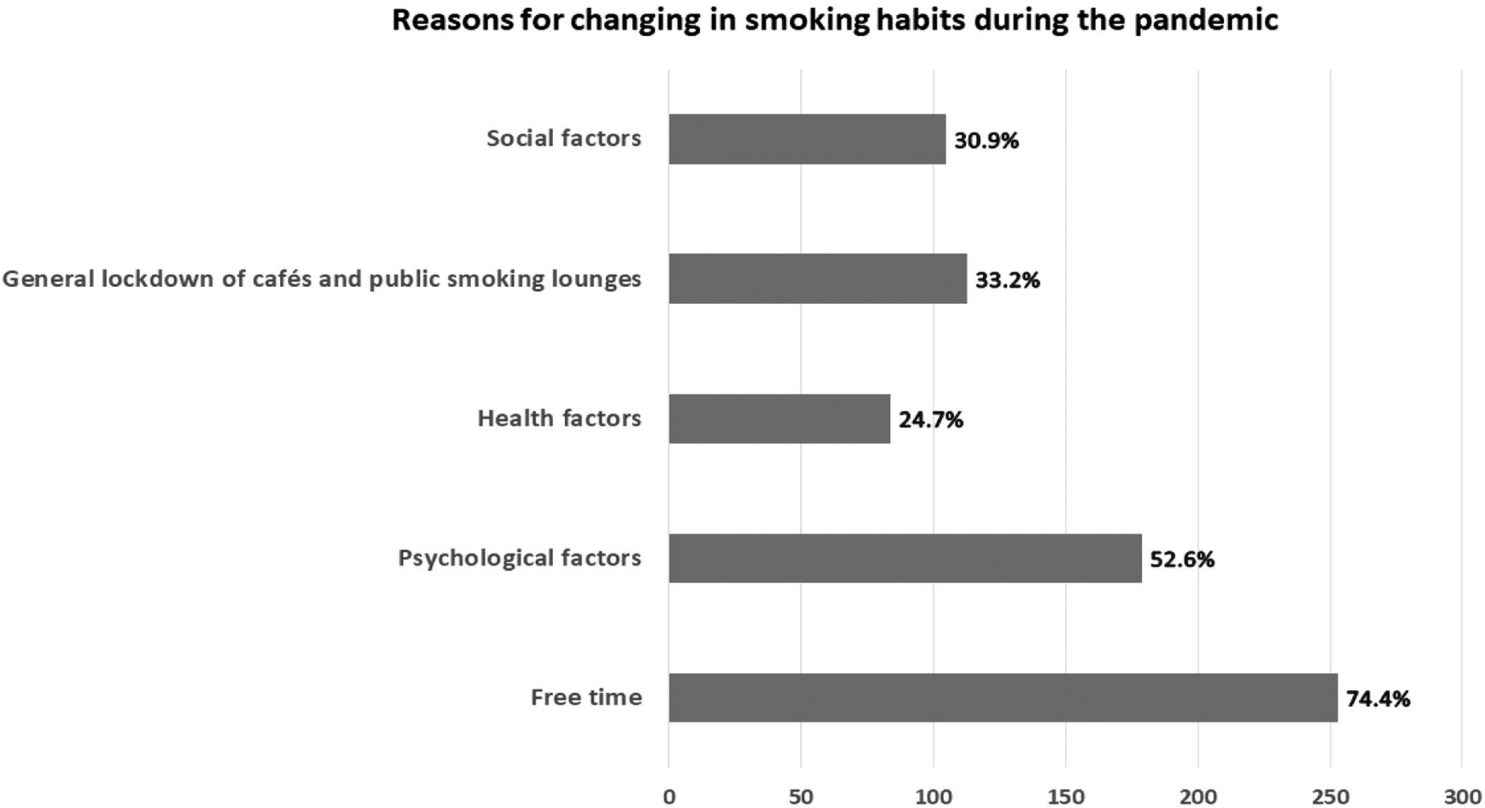
Reasons for changing in smoking habits during the pandemic among respondents who noticed any change in their smoking habits whether an increase or a decrease in the frequency of smoking (*n* = 340).

The current study showed that 253 (74.4%) of the respondents declared that the main cause for the change in the smoking habit was free time and boredom due to the general lockdown and quarantine. One hundred seventy‐nine (52.6%) reported that these changes were caused by psychological factors and different stressors that resulted from the financial burden of losing a job, income limitations, and online working or studying. Also, 113 (33.2%) linked the change in their smoking frequency with the general lockdown of cafés and public smoking lounges. While 105 (30.9%) declared that this change was caused by the influence of family members and friends. However, only 84 (24.7%) respondents realized that smoking has adverse effects on the immune system which is vital to combat diseases and their aftereffects like COVID‐19 (Figure 2).

## 4 DISCUSSION

Smoking is a worldwide health problem with a socioeconomic burden.^19^, ^20^, ^21^ Global efforts have been conducted to change this habit with no decline.^22^, ^23^ In the last two years, the COVID‐19 pandemic had an impact on human life including lifestyle, diet, economic, and health issues. Smoking habit is expected to be affected by COVID‐19 measures such as a lockdown. For instance, a study conducted in France showed that tobacco smoking increased during the COVID‐19 lockdown in the 14–34 years age group.^24^ Consistently, the current study, showed that the 18–35 age group individuals had a higher percentage of increased smoking in comparison with other age groups. However, there was no significant difference between gender and occupation and changes in smoking habits during the lockdown, which is consistent with the results conducted in Belgium.^25^

The current findings showed an increase in smoking habits among males during the COVID‐19 lockdown (43%) compared to the females' group (12%). This is consistent with an Italian study that showed an increase in male smokers (49.3%) during the COVID‐19 lockdown compared to a 22.2% increase in female smokers.^10^ In addition, the current study showed an increase in the smoking habit in 45.4% of the study population during the lockdown which is similar to the Italian study (36%). In this study, the individuals who declared changes in their smoking habits attributed these changes to monotony, increased free time, mental distress, and economic difficulties which is supporting the finding of the Italian study.^10^ Interestingly, the current results showed an increase in sleeping hours compared to a decrease in sleeping hours in the Italian Study. In addition, the current study is supporting the Italian findings about the reduction in the smoking habit among the 18–25 age group.^10^

In another study in Poland, 40% of smokers declared no change in their smoking level during the lockdown while 45.2% stated an increase in tobacco consumption.^26^ The current study is supporting the Poland study by showing that 28.9% of smokers had no changes in their smoking habit and 45.4% increased their smoking level. Interestingly, our findings also showed that gender and educational level were not significantly associated with changes in smoking levels during the lockdown. On the other hand, the current results showed that the 18–35 age group was significantly associated with changes in smoking habits which are not consistent with the Polish population.^26^ These differences can be attributed to different socioeconomic statuses, lifestyles, or cultural factors.

In a study of 679 participants conducted in Kuwait, 15% of males had an increased smoking rate during the pandemic^27^ compared to 21.2% of males in our study which can be attributed to the sample size, socioeconomic status, and lifestyles. In another Italian study, 3.3% of the study population declared that they have quit smoking and that the vast majority have decreased their smoking habits during the lockdown.^6^ On the other hand, a study of the English population found that 39.6% of the individuals had attempted smoking quitting during the lockdown,^28^ whereas the current study showed that 25.7% of the study population decreased their smoking habit or tried to quit during the lockdown.

In the aforementioned Belgian survey, the researchers found that 1%, 7.4%, and 2.5% of the total sample population had quit smoking, increased smoking, and decreased their smoking habits respectively. This means 6.4% of smokers quit during the lockdown, 48.1% declared an increase in their smoking habits, and 16.2% declared a decrease in their smoking habits.^25^ These results are very close to our results (45.4% increased, 28.9% tried to quit or decreased). Also, they found that increased smoking was associated with the younger age group which is consistent with our study. In addition, the current findings are supporting the Belgium study regarding the causes of more consumption during the lockdown including boredom, decreased socialization, psychological stressors, and work‐related changes.^25^ These are also consistent with a study from the United Kingdom of 132 smoker participants, those who experienced an increase in their smoking level described smoking as a coping mechanism to deal with psychological stressors such as anxiety, anger, and boredom.^29^

In a Greek study, they found a significant difference between increased smoking and age group; all age groups are associated with increased smoking except the young age group (18–29 years), and older age group (60–69 and ≥70 years).^30^ These results were different from the findings of the current study in which the highest percentage of smokers who increased their consumption of smoking during the pandemic was among (the 18–35 years) age group.

Another Italian study indicated that there were changes in cigarette smoking habits during the lockdown in 11.8% of the study population (7.7% increased and 4.1% decreased).^31^ Alike, the current study showed that 8.6% of the respondents increased their smoking habit and 4.8% decreased.

In a Croatian study, they found an overall increase in smoking consumption during the lockdown, and they also found a significant difference between females and the increase in daily cigarettes number during the lockdown.^32^ On the other hand, the current study found no significant difference between gender and changes in smoking habits.

A study from northern Italy of 105 smokers showed that 38% of smoker participants experienced an increase in smoking levels, which was associated with an increase in food consumption (60%).^33^ These results are close to our findings where 45.4% of smokers increased their smoking during the lockdown. This increase was associated with increased appetite, weight gain, more night meals, and more sugar and fast‐food intake in comparison with those who experienced less smoking during the lockdown.

## 5 CONCLUSION

The results of our study showed that the lockdown had a significant impact on people's lifestyles including smoking habits. Most of our sample's smoker participants experienced a change in their smoking level mostly, an increase. These changes were mostly attributed to the impact of the lockdown on people's life such as increased free time and boredom during the lockdown, financial difficulties, and other various stressors. In general, the study showed that smokers were more likely to adopt an unhealthy lifestyle during the lockdown in comparison with nonsmokers. The results showed that smokers adopted an unhealthy lifestyle represented by daily consumption of sugar and fast food and other unhealthy habits. While those who had a decrease in their smoking level experienced a somehow healthier lifestyle regarding nutrition and other aspects.

## 6 LIMITATIONS

This study tried to provide a general look at changes in people's habits, especially smoking during the worldwide pandemic crisis. But there were some limitations that we faced through this research. One of these limitations is that the study was conducted through an online survey with no possibility to verify data we collected from respondents, but this was the only possible method taking into consideration the extremely strict lockdown the country was put under during that time. In addition, males and less than 18 years old individuals are not fully represented in the current study.

## AUTHOR CONTRIBUTIONS

**Almu'atasim Khamees**: Conceptualization; data curation; formal analysis; writing—original draft; writing—review and editing. **Sajeda Awadi**: Conceptualization; data curation; formal analysis; writing—original draft; writing—review and editing. **Shireen Rawashdeh**: Writing—original draft. **Muna Talafha**: Writing—original draft. **Mai Alzoubi**: Writing—original draft. **Walaa Almdallal**: Writing—original draft. **Sharaf al‐Eitan**: Writing—review and editing. **Ahmad Saeed**: Writing—original draft. **Raed M. Al‐Zoubi**: Conceptualization; supervision; writing—review and editing. **Mazhar Salim Al‐Zoubi**: Conceptualization; data curation; formal analysis; supervision; validation; writing—original draft; writing—review and editing.

## CONFLICT OF INTEREST STATEMENT

The authors declare no conflict of interest.

## ETHICS STATEMENT

The research protocol was approved by the Institutional Review Board of Yarmouk University research ethics committee with the ethics approval no: IRB/2023/101.

## TRANSPARENCY STATEMENT

The lead author Raed M. Al‐Zoubi affirms that this manuscript is an honest, accurate, and transparent account of the study being reported; that no important aspects of the study have been omitted; and that any discrepancies from the study as planned (and, if relevant, registered) have been explained.

## Data Availability

All authors have read and approved the final version of the manuscript. The corresponding author had full access to all of the data in this study and takes complete responsibility for the integrity of the data and the accuracy of the data analysis. If you require the data, or if you wish to review the protocol, you can contact the authors via emails: ralzoubi@hamad.qa or mszoubi@yu.edu.jo.
